# Targeted Gene and Genome-Editing Strategies for Epilepsy: Experimental Advances and Translational Challenges

**DOI:** 10.3390/ijms27062845

**Published:** 2026-03-20

**Authors:** Bilal Ahmad Seh, Kashf Rafiq, Adam Legradi, Mohd Yaqub Mir

**Affiliations:** 1 Institute of Biochemistry and Biophysics, Polish Academy of Sciences, 02106 Warszawa, Poland; 2 Institute of Clinical Medicine, Faculty of Health Sciences, University of Eastern Finland, FI-70029 Kuopio, Finland; 3 Department of Cell Biology and Molecular Medicine, University of Szeged, H-6720 Szeged, Hungary; legradam@bio.u-szeged.hu; 4 Epilepsy Centre, Department of Clinical Sciences, Lund University Hospital, 221 00 Lund, Sweden

**Keywords:** epilepsy, gene therapy, genome editing, CRISPR technologies, drug-resistant epilepsy

## Abstract

Epilepsy affects more than 50 million individuals worldwide, and approximately one-third of patients remain refractory to existing antiseizure medications. Advances in gene therapy and genome editing have opened new possibilities for disease-modifying interventions that directly target the molecular and circuit-level mechanisms underlying epileptogenesis. Recent progress in central nervous system tropic viral vectors, non-viral delivery systems, and programmable genome-editing technologies has enabled precise manipulation of neuronal and glial function in preclinical epilepsy models. Strategies range from restoration of haploinsufficient genes implicated in monogenic epilepsies, such as SCN1A in Dravet syndrome, to modulation of neuronal excitability through engineered ion channels, neuropeptides, and astrocyte-based approaches. In parallel, CRISPR-derived platforms, including transcriptional activation and repression systems, base editing, and prime editing, offer new avenues for regulating gene expression in post-mitotic neurons without introducing double-strand DNA breaks. Despite these advances, significant translational challenges remain, including efficient and cell-type-specific delivery, long-term safety, and the risk of network-level side effects in the epileptic brain. This review critically examines recent gene therapy and genome-editing approaches for epilepsy, highlights key technological and biological barriers to clinical translation, and discusses emerging strategies that may enable durable and targeted treatments for drug-resistant epilepsies.

## 1. Introduction

Epilepsy is a chronic neurological disorder characterized by recurrent, unprovoked seizures resulting from pathological neuronal hyperexcitability and network synchronization. It affects more than 50 million individuals worldwide and encompasses a broad spectrum of etiologies, including genetic, structural, metabolic, immune, infectious, and idiopathic causes [1,2,3]. Despite the availability of numerous antiseizure medications, approximately one-third of patients develop drug-resistant epilepsy, a condition associated with increased morbidity, cognitive impairment, psychiatric comorbidities, and elevated mortality risk [4]. For these patients, surgical resection or neuromodulation may offer benefit, yet many are not eligible or fail to achieve durable seizure freedom, highlighting a critical unmet clinical need [5,6].

Gene therapy has emerged as a compelling therapeutic strategy for epilepsy because seizure disorders frequently arise from well-defined molecular and cellular dysfunctions that are amenable to genetic modulation. Advances in viral vector engineering, particularly the development of adeno-associated virus (AAV) platforms, have enabled long-term gene expression in post-mitotic neurons and glial cells with a favorable safety profile [7,8,9]. The clinical success of AAV-mediated gene therapies in neurological and neuromuscular disorders, including spinal muscular atrophy and inherited retinal dystrophies, has further accelerated interest in applying these technologies to epilepsy [10,11,12]. Both monogenic and acquired epilepsies present rational targets for gene-based interventions. However, it is important to emphasize that epilepsy encompasses a heterogeneous group of disorders with diverse etiologies, and gene-based therapeutic approaches are most applicable to specific epilepsy subtypes with clearly defined molecular mechanisms.

**Figure 1:**
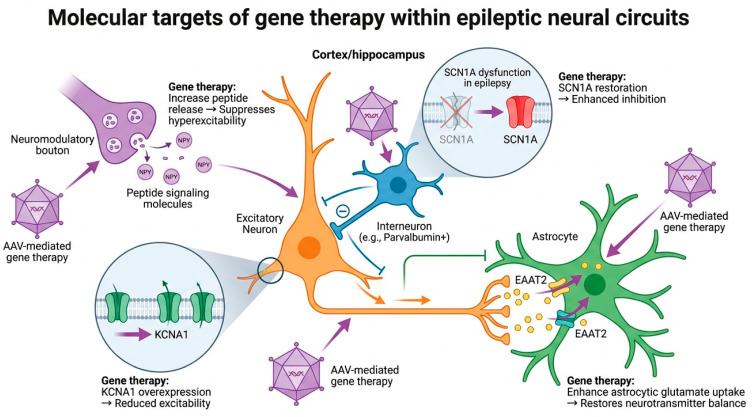
Molecular targets of gene therapy within epileptic neural circuits. Schematic illustration of gene therapy strategies targeting neuronal and glial mechanisms involved in epileptogenesis. These include modulation of neuronal excitability through potassium channel overexpression (KCNA1), restoration of inhibitory interneuron function via SCN1A gene regulation, neuromodulatory peptide signaling such as neuropeptide Y (NPY) to suppress hyperexcitability, and astrocyte-targeted approaches enhancing glutamate uptake through EAAT2. AAV-mediated gene delivery systems are commonly used to target these mechanisms within cortical and hippocampal circuits.

In monogenic epileptic encephalopathies, loss-of-function or gain-of-function variants in ion channel and synaptic genes directly disrupt neuronal excitability and inhibitory control [2,13]. Dravet syndrome, caused primarily by haploinsufficiency of the SCN1A gene encoding the Nav1.1 sodium channel, exemplifies this paradigm and has become a leading model for precision gene therapy approaches in epilepsy [14,15]. In acquired focal epilepsies, such as temporal lobe epilepsy, maladaptive changes in excitatory and inhibitory balance, neurotransmitter signaling, and glial homeostasis provide additional opportunities for targeted genetic modulation [5,16]. These molecular and cellular targets within epileptic neural circuits including ion channel modulation, interneuron dysfunction, neuromodulatory peptide signaling, and astrocyte-mediated neurotransmitter regulation are illustrated schematically in Figure 1.

Beyond classical gene replacement, genome-editing and gene-regulation technologies have fundamentally expanded the therapeutic landscape for epilepsy. CRISPR-based systems enable programmable manipulation of endogenous genes, allowing either disruption, correction, or transcriptional modulation without the need for full-length transgene delivery [17]. Importantly, transcriptional activation and repression platforms based on catalytically inactive Cas9 (CRISPRa/i) permit reversible and tunable regulation of neuronal genes without introducing double-strand DNA breaks, a critical safety consideration in post-mitotic neurons [15,18]. More recently, base-editing and prime-editing technologies have enabled precise correction of pathogenic point mutations with reduced genotoxic risk, further expanding the applicability of genome editing to developmental and epileptic encephalopathies [19,20,21].

Despite these advances, significant translational challenges remain for gene therapy in epilepsy. Efficient and cell-type specific delivery across the blood–brain barrier, long-term safety and immunogenicity, network-level consequences of sustained gene modulation, and the high cost of vector manufacturing all represent major obstacles to widespread clinical implementation [6,22]. Addressing these challenges will be essential to move epilepsy gene therapies from experimental interventions toward routine clinical practice.

In this review, we critically examine recent advances in gene therapy and genome-editing strategies for epilepsy, with a particular emphasis on delivery platforms, molecular targets, and genome-regulation technologies that have demonstrated efficacy in preclinical models. We discuss how biological constraints unique to the epileptic brain have shaped therapeutic design and highlight emerging approaches that may enable durable, precise, and safe treatments for drug-resistant epilepsies.

## 2. Vector Platforms for Epilepsy Gene Therapy

Effective gene therapy for epilepsy critically depends on delivery systems capable of achieving precise, durable, and cell-type specific transgene expression within the central nervous system (CNS) [5,9]. Unlike peripheral disorders, epilepsy presents unique challenges for gene delivery, including the blood–brain barrier, the predominance of post-mitotic target cells, and the need to modulate defined neuronal and glial populations without disrupting broader network stability [6,23]. Consequently, vector selection is not merely a technical consideration but a central determinant of therapeutic efficacy and safety [5].

### 2.1. Viral Vectors for CNS Delivery

Among available platforms, adeno-associated viruses (AAVs) have emerged as the most widely used vectors for epilepsy gene therapy due to their favorable safety profile, long-term transgene expression, and ability to transduce non-dividing neurons [7,8,9]. AAV-mediated gene delivery has been particularly successful in preclinical epilepsy models, where sustained modulation of neuronal excitability is required to achieve durable seizure suppression [15,16,24]. The development of CNS-tropic AAV serotypes and engineered capsids has further expanded their utility, enabling broader brain distribution and improved penetration across the blood–brain barrier [11,25]).

For epilepsy applications, vector tropism is a critical consideration. Many therapeutic strategies aim to selectively target inhibitory interneurons, astrocytes, or discrete excitatory neuronal populations, necessitating the use of cell-type-specific promoters or enhancer elements in combination with appropriate AAV serotypes [23,26]. For example, AAV9 and engineered variants have demonstrated robust transduction of neurons throughout the brain following systemic or intracerebroventricular delivery, while interneuron-selective regulatory elements have enabled preferential targeting of GABAergic circuits implicated in seizure generation [11]. However, widespread CNS transduction also raises concerns regarding off-target effects and excessive inhibition, underscoring the need for precise spatial and cellular control [5,6].

Cargo capacity remains an important limitation of AAV-based approaches, particularly for epilepsy-associated genes such as *SCN1A*, which exceed the packaging limits of single AAV vectors [15]. This constraint has driven the development of alternative strategies, including transcriptional activation of endogenous genes and dual-vector systems, although the latter suffer from reduced co-transduction efficiency [15,27]. These challenges have directly shaped the evolution of genome-regulation approaches in epilepsy, favoring methods that modulate native gene expression rather than relying on full-length gene replacement [6,15].

CNS-tropic AAV serotypes used in neurological gene therapy are summarized in Table 1.

### 2.2. Lentiviral Vectors and Stable Gene Integration

Lentiviral vectors offer the advantage of stable genomic integration and sustained transgene expression, making them attractive for chronic neurological disorders requiring long-term modulation [7,8]. In epilepsy research, lentiviral vectors have been extensively used in focal delivery paradigms, particularly in rodent models of temporal lobe epilepsy, where localized expression of therapeutic genes such as engineered potassium channels has produced prolonged seizure suppression [16,24]. The ability to deliver larger genetic payloads compared with AAV vectors further expands the range of potential targets.

Despite these advantages, the integration of lentiviral vectors into the host genome raises safety concerns, including insertional mutagenesis, which may limit their translational applicability for epilepsy [8,40]. While the development of self-inactivating lentiviral constructs has substantially improved safety profiles, the irreversible nature of genomic integration remains a consideration, particularly in disorders where fine-tuned regulation of neuronal activity is essential [41]. As a result, lentiviral approaches in epilepsy are currently viewed as powerful experimental tools and potential candidates for highly localized therapies rather than broadly deployable clinical platforms [5].

### 2.3. Non-Viral Delivery Systems

Non-viral delivery platforms, including lipid nanoparticles and polymer-based systems, have gained increasing attention as alternatives to viral vectors due to their reduced immunogenicity and expanded cargo capacity [42]. Lipid nanoparticles have demonstrated remarkable success in systemic nucleic acid delivery, and recent advances in ionizable lipid design have improved tissue specificity and intracellular release [21,43,44]. However, efficient and targeted delivery to the brain remains a major challenge, and most clinically validated lipid nanoparticle systems exhibit strong hepatic tropism rather than CNS selectivity [45,46].

In the context of epilepsy, non-viral systems hold particular promise for transient gene-modulation strategies, such as RNA-based therapies or genome-editing components delivered as ribonucleoprotein complexes [21,47]. These approaches may mitigate long-term safety concerns associated with permanent gene modification while enabling repeated dosing if necessary [22]. Nevertheless, current limitations in brain penetration, cellular uptake, and endosomal escape have thus far restricted their application primarily to preclinical studies [42,48].

### 2.4. Implications for Epilepsy-Focused Gene Therapy

Collectively, the strengths and limitations of existing vector platforms have profoundly influenced the design of gene therapy strategies for epilepsy [5,6]. Viral vectors, particularly AAVs, remain the leading candidates for clinical translation due to their efficiency and durability, while non-viral systems offer complementary opportunities for transient or modular interventions [9,47]. Importantly, the constraints imposed by vector biology have accelerated interest in genome-regulation approaches such as CRISPR-based transcriptional modulation that can achieve therapeutic effects within existing packaging limits [15,27]. As gene therapy for epilepsy continues to mature, advances in vector engineering, delivery precision, and cell-type specificity will be essential to enable safe and effective modulation of epileptic networks [6].

Although many delivery platforms were initially developed for systemic gene therapy applications, several of these technologies have recently been adapted for experimental epilepsy models and central nervous system gene delivery. Because epilepsy gene therapy requires efficient delivery to neuronal and glial populations within the central nervous system, the major viral and non-viral delivery platforms currently investigated for CNS applications are summarized in Table 2.

Key advantages and limitations of viral and non-viral delivery platforms are compared in Table 2.

## 3. Genome Editing and Gene Regulation Strategies in Epilepsy

### 3.1. Rationale for Genome Editing in Epilepsy

**Figure 2:**
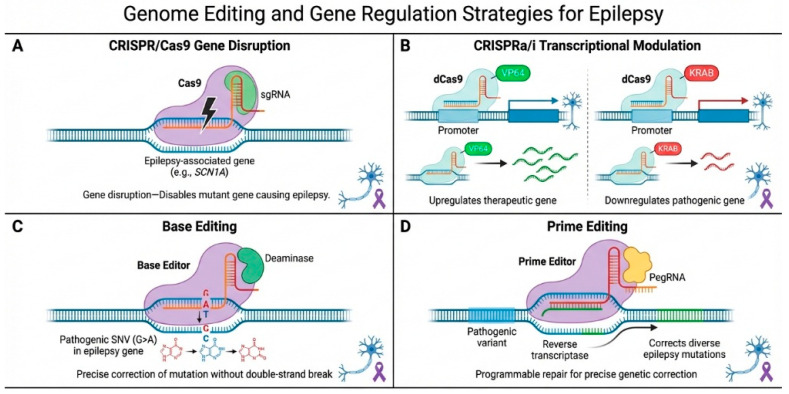
Genome-editing and gene-regulation strategies for epilepsy. Schematic overview of programmable genome-engineering approaches with potential therapeutic applications in epilepsy. (**A**) CRISPR/Cas9-mediated gene disruption generates targeted double-strand DNA breaks leading to gene knockout through non-homologous end joining. (**B**) CRISPRa/i systems use catalytically inactive Cas9 (dCas9) fused to transcriptional activators (e.g., VP64) or repressors (e.g., KRAB) to modulate endogenous gene expression without DNA cleavage. (**C**) Base editing enables precise single-nucleotide substitutions through Cas9-nickase fused to a deaminase enzyme. (**D**) Prime editing uses a Cas9-nickase–reverse transcriptase fusion and prime editing guide RNA (pegRNA) to introduce targeted insertions, deletions, or substitutions without generating double-strand breaks.

While viral vector-mediated gene delivery has enabled durable transgene expression in the central nervous system, classical gene replacement strategies face inherent limitations in epilepsy, including restricted cargo capacity, lack of endogenous regulatory control, and the risk of excessive or ectopic gene expression. These constraints are particularly relevant in the epileptic brain, where precise modulation of neuronal excitability is essential to avoid network destabilization [5,6]. Genome-editing and gene-regulation technologies address many of these challenges by enabling direct manipulation of endogenous genes, allowing therapeutic effects to be achieved without full-length transgene delivery [17,25]. The principal genome-editing and gene-regulation strategies currently being explored for epilepsy including CRISPR/Cas9-mediated gene disruption, CRISPR-based transcriptional activation or repression (CRISPRa/i), base editing, and prime editing are summarized schematically in Figure 2.

Epilepsy is especially well suited for genome-based interventions because many disease mechanisms arise from discrete alterations in ion channels, neurotransmitter systems, and synaptic regulators that govern excitatory inhibitory balance. Importantly, partial restoration or modulation of gene expression is often sufficient to produce meaningful seizure suppression, reducing the need for complete genetic correction [15,16].

### 3.2. CRISPR/Cas9-Mediated Gene Disruption

The CRISPR/Cas9 system enables targeted DNA cleavage guided by a programmable single-guide RNA, resulting in gene disruption through error-prone non-homologous end joining (NHEJ) or precise sequence correction via homology-directed repair (HDR) [17,49]. In epilepsy research, CRISPR/Cas9-mediated gene disruption has been used to interrogate and therapeutically target genes that promote epileptogenesis.

A recent study demonstrated that neuron-specific AAV-mediated deletion of the Alox5 gene significantly reduced seizure severity and progression in mouse models of temporal lobe epilepsy [50]. In addition to suppressing spontaneous seizures, Alox5 deletion ameliorated cognitive deficits and reduced neuropathological features such as neuronal loss and astrogliosis. Mechanistically, this intervention decreased intracellular calcium signaling and reduced glutamate release through inhibition of CAMKII-dependent synaptic pathways [50]. These findings highlight the potential of CRISPR/Cas9-mediated gene disruption as a disease-modifying strategy for acquired epilepsies.

However, permanent gene disruption raises important safety considerations, particularly for genes expressed across multiple cell types or involved in essential physiological processes. Furthermore, HDR-based correction is inefficient in post-mitotic neurons, limiting the feasibility of precise sequence replacement in the adult brain [18,51]. These challenges have driven interest in alternative CRISPR-based approaches that avoid double-strand DNA breaks.

### 3.3. CRISPR-Based Transcriptional Regulation (CRISPRa and CRISPRi)

Catalytically inactive Cas9 (dCas9) fused to transcriptional activators or repressors enables programmable upregulation or downregulation of endogenous gene expression without inducing DNA cleavage [18]. This approach is particularly attractive for epilepsy, where reversible and tunable modulation of neuronal genes can restore network balance while minimizing genotoxic risk.

CRISPRa-based upregulation of Scn1a has emerged as one of the most compelling genome-regulation strategies for epilepsy. In Dravet syndrome, haploinsufficiency of SCN1A leads to impaired function of inhibitory GABAergic interneurons and severe, drug-resistant seizures [13,14]. Colasante et al. demonstrated that AAV-delivered dCas9-based transcriptional activation selectively increased Scn1a expression in GABAergic interneurons, restoring Nav1.1 protein levels and rescuing interneuron excitability in both cellular and mouse models of Dravet syndrome [15]. This intervention significantly reduced seizure frequency and improved survival, even when administered after symptom onset.

Importantly, CRISPRa-mediated Scn1a upregulation bypasses the AAV cargo limitations that preclude full-length SCN1A gene replacement and preserves endogenous regulatory architecture. Moreover, because dCas9 does not induce DNA cleavage, this approach minimizes the risk of permanent off-target mutations in post-mitotic neurons [15]. Nonetheless, current implementations often rely on dual-AAV systems, which suffer from limited co-transduction efficiency and pose challenges for clinical translation.

CRISPR-based transcriptional modulation has also been applied to acquired epilepsies. CRISPRa-mediated upregulation of Kcnal (encoding the Kv1.1 potassium channel) reduced seizure frequency and severity in rodent models of temporal lobe epilepsy, demonstrating that endogenous ion channel modulation can effectively suppress neuronal hyperexcitability [15,16]. Together, these studies establish CRISPRa and CRISPRi as versatile tools for fine-tuning epileptic networks.

### 3.4. Base Editing and Prime Editing

Base editing and prime editing represent next-generation genome-editing technologies that enable precise nucleotide modifications without generating double-strand DNA breaks [19,20]. Base editors catalyze targeted single-base conversions, while prime editors enable small insertions, deletions, and substitutions through reverse transcription-mediated editing guided by prime editing guide RNAs (pegRNAs).

These technologies are particularly relevant for genetic epilepsies caused by point mutations, where correction of a single nucleotide may restore sufficient gene function to ameliorate disease phenotypes. Base and prime editing have demonstrated efficacy in correcting pathogenic mutations in several neurological and neuromuscular disorders, including Huntington’s disease, Duchenne muscular dystrophy, and inherited retinal diseases, providing proof-of-concept for their application in epilepsy [52,53,54,55].

Despite their promise, significant challenges remain for applying base and prime editing in the epileptic brain. Efficient delivery to widespread neuronal populations, control of off-target deamination, and optimization of editing efficiency in post-mitotic neurons remain active areas of investigation [21,51]. Nevertheless, ongoing advances in editor design and delivery platforms continue to expand their translational potential.

Despite their considerable promise, genome-editing strategies face several technical constraints when applied to the central nervous system. Efficient editing in post-mitotic neurons remains challenging, as DNA repair pathways such as homology-directed repair are largely inactive in mature neuronal populations. In addition, delivery of genome-editing machinery is constrained by the limited packaging capacity of AAV vectors, which can complicate the delivery of larger editing systems such as base editors and prime editors that exceed standard vector size limits. Another emerging concern relates to the potential immunogenicity of bacterial Cas proteins, including Cas9, which may trigger host immune responses that limit long-term expression or reduce therapeutic efficacy [24,36]. These factors highlight the importance of continued efforts to develop compact genome-editing systems and improved delivery strategies tailored to the unique biology of the nervous system.

### 3.5. Implications for Clinical Translation

Collectively, genome-editing and gene-regulation technologies have transformed the therapeutic landscape for epilepsy by enabling precise, mechanism-driven interventions that extend beyond conventional gene replacement. CRISPRa-based transcriptional modulation currently represents the most clinically tractable approach, offering a balance between efficacy, safety, and vector compatibility. As delivery technologies mature and editing tools become more efficient and specific, genome-based therapies are poised to play a central role in the development of durable treatments for drug-resistant epilepsies.

Distinct CRISPR-based genome editing and gene regulation strategies relevant to epilepsy are summarized in Table 3.

## 4. Mechanism-Based Gene Therapy Strategies for Epilepsy

### 4.1. Targeting Neuronal Excitability Through Ion Channel Modulation

A central pathophysiological feature of epilepsy is an imbalance between neuronal excitation and inhibition that promotes hypersynchronous firing and seizure propagation. Consequently, gene therapy strategies aimed at restoring inhibitory control or reducing intrinsic neuronal excitability have shown considerable promise across multiple epilepsy models [1,5]. Among these approaches, modulation of potassium channel activity has emerged as a particularly effective mechanism for long-term seizure suppression.

The voltage-gated potassium channel Kv1.1, encoded by KCNA1, plays a key role in regulating action potential threshold and repetitive firing. Reduced Kv1.1 function has been associated with increased neuronal excitability and epileptiform activity. Lentiviral delivery of engineered Kcna1 constructs into epileptogenic brain regions produced sustained seizure suppression in rodent models of temporal lobe epilepsy [24]. Subsequent studies using AAV-mediated Kcna1 overexpression confirmed these findings, demonstrating reduced seizure frequency and duration without overt neurotoxicity [16]. Importantly, genome-regulation approaches have further supported this strategy. For example, CRISPRa-mediated activation of *Scn1a* restored inhibitory interneuron excitability and reduced seizures in Dravet syndrome models [15], while increased expression of potassium channels such as *Kcna1* has been shown to suppress neuronal hyperexcitability in experimental epilepsy models [16,26]. These studies collectively highlight ion channel modulation as a robust disease-modifying strategy for epilepsy and underscore the advantage of approaches that preserve endogenous regulatory control over neuronal excitability. However, important translational considerations remain, including optimal AAV serotype selection, promoter specificity, and vector dosing required to achieve sustained therapeutic expression without altering normal neuronal network function.

### 4.2. Restoring Inhibitory Neurotransmission in Genetic Epilepsies

Genetic epilepsies caused by loss-of-function mutations in genes essential for inhibitory interneuron function represent particularly compelling candidates for precision gene therapy. Dravet syndrome, most commonly caused by haploinsufficiency of SCN1A, results in impaired Nav1.1 channel expression in GABAergic interneurons and severe, early-onset epilepsy [13,14].

Recent preclinical studies have demonstrated that targeted upregulation of Scn1a expression in inhibitory interneurons can effectively rescue disease phenotypes. An AAV9-delivered engineered transcription factor designed to selectively enhance Scn1a transcription in GABAergic neurons significantly reduced spontaneous seizures and improved survival in mouse models of Dravet syndrome [56]. Similarly, dCas9-based CRISPRa approaches restored Nav1.1 expression, rescued interneuron excitability, and attenuated both spontaneous and hyperthermia-induced seizures [15]. These strategies circumvent the packaging limitations of AAV vectors and avoid the risks associated with permanent genome disruption.

Together, these findings establish interneuron-specific gene regulation as a highly effective and mechanistically precise approach for treating monogenic epileptic encephalopathies.

### 4.3. Neuropeptide-Based Suppression of Excitatory Neurotransmission

Beyond ion channels, neuromodulatory peptides have been extensively explored as gene therapy targets for epilepsy due to their ability to suppress excitatory neurotransmission and modulate synaptic plasticity. Neuropeptide Y (NPY) is one of the most extensively studied candidates, acting through multiple receptor subtypes to modulate excitatory neurotransmission and synaptic plasticity in epileptic networks [50,51]. Direct genetic delivery of NPY to epileptogenic brain regions consistently reduced spontaneous seizure frequency in rodent models of temporal lobe epilepsy and kainate-induced seizures [52,53,54]. Moreover, AAV-mediated co-expression of NPY with its cognate receptors further enhanced anticonvulsant efficacy, producing seizure reductions of up to 45% in preclinical models [57,58,59]. Additional neuromodulatory peptides, including galanin and somatostatin, have also demonstrated seizure-suppressive effects when delivered via viral vectors [60,61].

While these approaches robustly suppress seizures, their broader effects on cognition and synaptic plasticity remain an important consideration. Some studies report minimal cognitive impairment, whereas others suggest potential adverse effects on learning and memory, highlighting the need for precise spatial and temporal control of peptide expression [62,63].

### 4.4. Astrocyte-Targeted Gene Therapy and Metabolic Modulation

Astrocytes play a critical role in regulating extracellular neurotransmitter levels, ion homeostasis, and metabolic support within the epileptic brain. Dysregulation of astrocytic function contributes to seizure initiation and maintenance, making glial cells attractive targets for gene therapy [5].

One prominent astrocyte-based strategy involves suppression of adenosine kinase (ADK), an enzyme that metabolizes adenosine, a potent endogenous anticonvulsant. AAV-mediated, microRNA-driven knockdown of Adk in astrocytes increased extracellular adenosine levels and significantly reduced seizure duration and severity in rodent epilepsy models [64]. This approach highlights the potential of targeting non-neuronal components of epileptic networks to achieve seizure control.

Similarly, astrocytic delivery of therapeutic proteins such as glial cell line-derived neurotrophic factor (GDNF) has been shown to suppress seizures and modify disease progression in temporal lobe epilepsy models [65]. These findings reinforce the concept that epilepsy gene therapy need not be restricted to neurons, and that modulation of the neuroglial environment can yield meaningful therapeutic benefits.

Beyond adenosine metabolism and neurotrophic signaling, additional astrocyte-mediated mechanisms contribute significantly to epileptogenesis and represent potential targets for gene therapy. Astrocytes regulate extracellular glutamate concentrations through high-affinity transporters, particularly excitatory amino acid transporter-2 (EAAT2), also known as GLT-1. Reduced GLT-1 expression has been consistently observed in both experimental and human temporal lobe epilepsy and is associated with impaired glutamate clearance and increased excitotoxic neuronal activity [66]. Restoration or enhancement of GLT-1 expression has therefore been proposed as a potential therapeutic strategy to stabilize synaptic glutamate homeostasis and suppress seizure propagation.

Astrocytes also play a central role in extracellular potassium buffering through inwardly rectifying potassium channels such as Kir4.1. Astrocytic potassium buffering mediated by inwardly rectifying potassium channels such as Kir4.1 plays an important role in maintaining extracellular potassium homeostasis in the brain, and impaired potassium clearance has been linked to increased neuronal hyperexcitability and seizure susceptibility in epilepsy [67]. Gene therapy approaches aimed at restoring astrocytic potassium buffering capacity may therefore represent an additional avenue for modulating network excitability in epilepsy.

Targeted expression of therapeutic genes in astrocytes can be achieved using astrocyte-specific promoters such as the glial fibrillary acidic protein (GFAP) promoter, which has been widely used to restrict viral transgene expression to astrocytic populations in experimental gene therapy studies [68]. Incorporating astrocyte-specific regulatory elements may therefore enhance the safety and precision of gene therapy interventions targeting neuroglial mechanisms in epilepsy.

However, astrocytes exhibit substantial regional and functional heterogeneity within the brain, and broad modulation of astrocytic signaling pathways may produce context-dependent effects on neuronal networks that remain incompletely understood. Further investigation will therefore be necessary to determine the long-term safety and therapeutic potential of astrocyte-targeted gene therapies in epilepsy.

Representative mechanism-based gene therapy strategies for epilepsy are summarized in Table 4.

### 4.5. Summary of Mechanism-Based Approaches

Mechanism-driven gene therapy strategies have demonstrated robust seizure suppression across a range of genetic and acquired epilepsy models. Approaches targeting ion channels, inhibitory interneurons, neuromodulatory peptides, and astrocytic metabolism illustrate the diversity of molecular entry points available for therapeutic intervention. Importantly, these strategies emphasize modulation of network function rather than complete genetic correction, aligning well with the biological realities of the epileptic brain and the constraints of current delivery technologies.

## 5. Translational and Clinical Challenges in Epilepsy Gene Therapy

### 5.1. Delivery Constraints and Cell-Type Specificity

Efficient and selective delivery of gene therapies to relevant brain regions and cell populations remains one of the most significant barriers to clinical translation in epilepsy. The blood–brain barrier (BBB) severely restricts systemic delivery of both viral and non-viral vectors, often necessitating intracerebral, intrathecal, or intracerebroventricular administration routes [9,23]. While engineered AAV capsids with enhanced CNS tropism have improved brain-wide transduction in preclinical models, their performance in large animals and humans remains less predictable [11,25].

Cell-type specificity is particularly critical in epilepsy, where indiscriminate modulation of neuronal excitability can exacerbate seizures or impair cognitive function. Many therapeutic strategies require selective targeting of inhibitory interneurons or astrocytes to restore network balance without inducing excessive inhibition [5,26]. Although cell-type specific promoters and enhancers have improved targeting precision, variability in expression levels and regional specificity continues to pose challenges for clinical scalability.

### 5.2. Timing of Intervention and Disease Heterogeneity

The timing of gene therapy administration represents another major translational hurdle. Many genetic epilepsies manifest early in development, raising the question of whether therapeutic intervention must occur during critical neurodevelopmental windows to achieve maximal benefit [13,14]. Preclinical studies have demonstrated that early intervention can yield robust seizure suppression and improved survival; however, neonatal or early postnatal delivery poses ethical, logistical, and regulatory challenges in human patients [10,15].

In contrast, acquired epilepsies such as temporal lobe epilepsy often develop after an initial insult, with seizures emerging following a latent epileptogenic phase. This heterogeneity complicates patient stratification and therapeutic targeting, as molecular drivers may differ across disease stages and individuals [2,5]. Consequently, therapies effective in one epilepsy subtype may not generalize across the broader patient population.

### 5.3. Long-Term Safety and Network-Level Effects

Sustained modulation of neuronal gene expression raises important safety considerations, particularly in the context of lifelong diseases such as epilepsy. Persistent overexpression or suppression of ion channels and neurotransmitter systems may alter synaptic plasticity, cognitive function, or network homeostasis over time [62,63]. Although many preclinical studies report favorable safety profiles, long-term follow-up data remain limited.

Genome-editing approaches introduce additional safety concerns. While CRISPRa and CRISPRi systems avoid double-strand DNA breaks, off-target transcriptional effects and unintended alterations in gene regulatory networks must be carefully evaluated [18]. For permanent gene disruption strategies, such as CRISPR/Cas9-mediated knockouts, the irreversibility of genomic changes necessitates rigorous assessment of both on-target and off-target consequences, particularly when targeting genes expressed in multiple cell types [50,72].

In addition to conventional safety considerations, gene therapies targeting neuronal excitability must also account for potential network-level consequences. Because epilepsy arises from dysregulated neuronal circuits rather than isolated molecular defects, sustained modification of ion channels or neurotransmitter systems could theoretically alter synaptic plasticity or cognitive processes over time. For example, excessive suppression of neuronal excitability could impair normal information processing, while widespread expression of therapeutic transgenes might disrupt regional network dynamics. These concerns highlight the importance of spatially restricted delivery strategies and cell-type-specific promoters to minimize unintended network perturbations.

### 5.4. Immune Responses and Repeat Dosing

Host immune responses to viral vectors represent another significant obstacle to durable gene therapy. Pre-existing neutralizing antibodies against AAV capsids can reduce transduction efficiency and limit patient eligibility, while adaptive immune responses may compromise long-term expression or preclude repeat dosing [22,73]. These concerns are particularly relevant for epilepsy, where disease progression or incomplete initial efficacy may necessitate additional interventions.

Strategies to mitigate immune responses, including capsid engineering, immunosuppression, and alternative delivery routes, are under active investigation, but their long-term effectiveness and safety remain to be established [9,41].

### 5.5. Manufacturing, Cost, and Regulatory Considerations

The high cost of gene therapy development and manufacturing poses substantial challenges for widespread clinical adoption. Complex vector production processes, stringent quality control requirements, and limited scalability contribute to treatment costs that may exceed those of conventional therapies by several orders of magnitude. These economic barriers raise concerns regarding equitable access, particularly for patients in low- and middle-income countries.

Regulatory pathways for CNS-directed gene therapies and genome-editing approaches are still evolving, with additional scrutiny applied to interventions involving permanent genetic modification or early-life administration. Establishing standardized outcome measures, including reliable biomarkers of seizure control and disease modification, will be essential for accelerating clinical translation [5,6].

### 5.6. Summary of Translational Challenges

Collectively, challenges related to delivery, timing, safety, immune responses, and cost continue to constrain the clinical translation of epilepsy gene therapies. Addressing these barriers will require coordinated advances in vector engineering, genome-editing precision, patient stratification, and regulatory frameworks. Importantly, many of these challenges are not unique to epilepsy, suggesting that progress in this field will have broad implications for gene therapy across neurological disorders.

## 6. Future Directions and Outlook

The rapid evolution of gene therapy and genome-editing technologies has positioned epilepsy as one of the most promising neurological indications for durable, mechanism-driven genetic interventions. Continued progress in this field will depend on addressing key technological and biological limitations while refining strategies that align with the complex network dynamics of the epileptic brain.

Although gene therapy and genome-editing approaches for epilepsy remain largely at the preclinical stage, recent clinical successes of AAV-mediated gene therapies in other neurological disorders demonstrate the feasibility of gene delivery to the central nervous system. For example, AAV-based therapies have been successfully approved for conditions such as spinal muscular atrophy and inherited retinal dystrophies, providing important proof-of-concept for the clinical translation of CNS-directed gene therapies [12,37,74]. These advances support the potential future development of gene-based interventions targeting specific molecular mechanisms underlying epilepsy.

One major area of future development lies in improving delivery precision and efficiency. Advances in AAV capsid engineering, including structure-guided evolution and selection in large-animal models, are expected to yield vectors with improved brain penetration, reduced immunogenicity, and enhanced cell-type specificity [11,25]. Parallel efforts to develop compact genome-editing systems, including smaller Cas9 orthologs and optimized transcriptional regulators, may enable single-vector delivery of CRISPR-based therapeutics, overcoming current limitations associated with dual-AAV approaches [75,76].

Genome-regulation strategies are likely to play an increasingly central role in epilepsy therapy. CRISPRa and CRISPRi platforms offer reversible and tunable modulation of endogenous gene expression, an advantage in disorders where partial correction is sufficient to restore network balance [15,16]. As editing tools become more precise, base editing and prime editing may expand therapeutic options for monogenic epilepsies caused by point mutations, particularly if delivery and editing efficiency in post-mitotic neurons continue to improve [19,20,21].

Another important direction involves integrating gene therapy with improved disease stratification and biomarker development. Advances in genetic diagnostics and neurophysiological monitoring are enabling more precise classification of epilepsy subtypes, which will be essential for selecting appropriate gene-based interventions and defining meaningful clinical endpoints [2,6]. In this context, the identification of reliable biomarkers for disease modification beyond seizure frequency alone will be critical for evaluating long-term therapeutic impact.

Looking forward, combination approaches that integrate gene therapy with pharmacological treatment, neuromodulation, or cell-based strategies may further enhance efficacy while mitigating risks. Targeting both neuronal and glial components of epileptic networks, or combining genome regulation with neuroprotective or anti-inflammatory interventions, represents a promising avenue for future investigation [5,50].

In conclusion, gene therapy and genome editing have transitioned from experimental concepts to realistic therapeutic strategies for epilepsy. While significant challenges remain, continued innovation in vector design, genome-editing precision, and translational methodology suggests that gene-based interventions may ultimately provide durable and disease-modifying treatments for patients with drug-resistant epilepsies. Although these approaches hold considerable promise, gene therapy and genome-editing strategies for epilepsy remain largely experimental and require extensive preclinical validation and carefully designed clinical trials to establish their long-term safety and efficacy.

## Figures and Tables

**Table 1:** Adeno-associated virus (AAV) serotypes, tissue tropism, and therapeutic applications.

AAV Serotype	Primary Target Tissues	Representative Therapeutic Applications	References
AAV1	Skeletal muscle, heart	Muscular dystrophies, cardiac gene therapy	[28,29]
AAV2	Retina	Inherited retinal dystrophies (e.g., LCA)	[30]
AAV6	Skeletal muscle, CNS	Neuromuscular disorders (e.g., SMA)	[31,32]
AAV8	Liver	Hemophilia, metabolic liver diseases	[33,34,35]
AAV9	CNS, muscle	SMA, ALS, neurodegenerative diseases	[36,37,38]
AAV-rh10	CNS (primates)	Large-animal CNS gene therapy	[39]

**Table 2:** Comparison of viral and non-viral gene delivery platforms.

Delivery Platform	Cargo Type	Integration	CNS Relevance	Key Advantages	Key Limitations	Representative CNS/Epilepsy Applications	References
AAV vectors	DNA	No (episomal)	Efficient neuronal and glial transduction in CNS	Long-term expression, relatively low immunogenicity, strong neuronal tropism	Limited cargo capacity (~4.7 kb), pre-existing immunity, limited re-dosing	Gene therapy targeting SCN1A, KCNA1, neuropeptides (NPY), and astrocytic pathways in experimental epilepsy models	[9,11,15,16]
Lentiviral vectors	RNA (integrated as DNA)	Yes	Localized CNS delivery to neurons and glia	Stable genomic integration, larger cargo capacity than AAV	Risk of insertional mutagenesis, limited diffusion from injection site	Focal delivery of potassium channel genes (KCNA1) and other modulators of neuronal excitability in rodent epilepsy models	[7,26]
Lipid nanoparticles (LNPs)	mRNA, siRNA, RNP complexes	No	Experimental CNS delivery under development	Non-viral delivery, transient expression, repeat dosing possible	Limited blood–brain barrier penetration, liver tropism	Experimental delivery of RNA-based therapeutics and genome-editing components for neurological diseases	[37,38,41]
Polymeric nanoparticles	DNA, RNA	No	Experimental localized CNS delivery	Design flexibility, reduced immunogenicity	Lower transfection efficiency compared with viral vectors	Investigational gene delivery systems for localized brain gene therapy	[37,42]

**Table 3:** Comparison of CRISPR-based genome editing and gene regulation strategies.

Editing Platform	Molecular Mechanism	Type of Genetic Modification	Double-Strand Breaks	Key Advantages	Key Limitations	Representative Epilepsy/CNS Applications	References
CRISPR/Cas9	RNA-guided Cas9 nuclease introduces targeted DNA cleavage	Gene disruption or targeted sequence modification via NHEJ or HDR	Yes	Highly efficient genome editing, versatile targeting	Risk of off-target mutations, double-strand break toxicity, HDR inefficient in post-mitotic neurons	Experimental targeting of epilepsy-associated genes and investigation of epileptogenic pathways in animal models	[17,44,45]
CRISPRa/CRISPRi (dCas9)	Catalytically inactive Cas9 fused to transcriptional activators (e.g., VP64) or repressors (e.g., KRAB)	Transcriptional activation or repression of endogenous genes	No	Reversible gene regulation, avoids DNA cleavage, preserves endogenous gene regulation	Requires sustained expression, large cargo size often requiring dual-AAV delivery	Upregulation of SCN1A in Dravet syndrome models and modulation of neuronal excitability genes	[15,20]
Base editing	Cas9 nickase fused to cytidine or adenine deaminase enzymes	Single-nucleotide substitutions without double-strand breaks	No	Precise point mutation correction, suitable for post-mitotic neurons	Limited to specific base conversions, risk of bystander editing	Potential correction of pathogenic variants in genetic epilepsies caused by point mutations	[21,46]
Prime editing	Cas9 nickase fused to reverse transcriptase guided by prime editing guide RNA (pegRNA)	Insertions, deletions, and all base substitutions	No	Broad editing scope without double-strand breaks	Large editor size complicates viral delivery, relatively lower editing efficiency	Experimental correction of pathogenic mutations relevant to neurological disorders	[22,23]

**Table 4:** Gene therapy strategies for epilepsy: targets, approaches, and outcomes.

Target Gene/Pathway	Therapeutic Strategy	Vector/Platform	Epilepsy Model/Indication	Key Outcomes	References
SCN1A	Transcriptional augmentation (gene regulation)	AAV9, engineered transcription factor (ETX101)	Dravet syndrome	Reduced spontaneous seizures, improved survival	[56]
SCN1A	CRISPR activation (dCas9-based)	Dual AAV (dCas9-VP64 system)	Dravet syndrome (mouse models)	Restored interneuron excitability, reduced seizures	[15]
KCNA1 (Kv1.1)	Ion channel overexpression	Lentiviral vector/AAV	Focal and temporal lobe epilepsy	Sustained seizure suppression	[16,24]
Scn1A	CRISPR activation of endogenous gene	AAV-delivered CRISPRa	Temporal lobe epilepsy	Reduced seizure frequency, improved cognition	[15]
NPY	Neuropeptide overexpression	AAV	Temporal lobe epilepsy, generalized epilepsy	40% reduction in seizure frequency	[69,70]
NPY + Y2/Y5 receptors	Combined peptide and receptor expression	AAV	Kainate-induced epilepsy	Enhanced seizure suppression (31–45%)	[57,58]
GAD67	Increased GABA synthesis	AAV	Temporal lobe epilepsy	Reduced seizure frequency, delayed epileptogenesis	[71]
Adenosine kinase (ADK)	Astrocyte-targeted suppression	AAV-miRNA	Kainate-induced epilepsy	Increased adenosine, reduced seizure duration	[64]

## Data Availability

No new data were created or analyzed in this study. Data sharing is not applicable to this article.

## References

[1] Stafstrom C.E., Carmant L. (2015). Seizures and epilepsy: An overview for neuroscientists. Cold Spring Harb. Perspect. Med..

[2] Perucca P., Bahlo M., Berkovic S.F. (2020). The Genetics of Epilepsy. Annu. Rev. Genomics Hum. Genet..

[3] Thijs R.D., Surges R., O’Brien T.J., Sander J.W. (2019). Epilepsy in adults. Lancet.

[4] Sheng J., Liu S., Qin H., Li B., Zhang X. (2018). Drug-Resistant Epilepsy and Surgery. Curr. Neuropharmacol..

[5] Simonato M., Bennett J., Boulis N.M., Castro M.G., Fink D.J., Goins W.F., Gray S.J., Lowenstein P.R., Vandenberghe L.H., Wilson T.J. (2013). Progress in gene therapy for neurological disorders. Nat. Rev. Neurol..

[6] Street J.S., Qiu Y., Lignani G. (2023). Are Genetic Therapies for Epilepsy Ready for the Clinic?. Epilepsy Curr..

[7] Kay M.A., Glorioso J.C., Naldini L. (2001). Viral vectors for gene therapy: The art of turning infectious agents into vehicles of therapeutics. Nat. Med..

[8] Naldini L. (2015). Gene therapy returns to centre stage. Nature.

[9] Hudry E., Vandenberghe L.H. (2019). Therapeutic AAV Gene Transfer to the Nervous System: A Clinical Reality. Neuron.

[10] Mendell J.R., Sahenk Z., Al-Zaidy S., Rodino-Klapac L.R., Lowes L.P., Alfano L.N., Berry K., Miller N., Yalvac M., Dvorchik I. (2017). Follistatin Gene Therapy for Sporadic Inclusion Body Myositis Improves Functional Outcomes. Mol. Ther..

[11] Deverman B.E., Pravdo P.L., Simpson B.P., Kumar S.R., Chan K.Y., Banerjee A., Wu W.L., Yang B., Huber N., Pasca S.P. (2016). Cre-dependent selection yields AAV variants for widespread gene transfer to the adult brain. Nat. Biotechnol..

[12] Russell S., Bennett J., Wellman J.A., Chung D.C., Yu Z.F., Tillman A., Wittes J., Pappas J., Elci O., McCague S. (2017). Efficacy and safety of voretigene neparvovec (AAV2-hRPE65v2) in patients with RPE65-mediated inherited retinal dystrophy: A randomised, controlled, open-label, phase 3 trial. Lancet.

[13] Lopez-Santiago L., Isom L.L. (2019). Dravet Syndrome: A Developmental and Epileptic Encephalopathy. Epilepsy Curr..

[14] Gataullina S., Dulac O. (2018). Is epilepsy the cause of comorbidities in Dravet syndrome?. Dev. Med. Child Neurol..

[15] Colasante G., Lignani G., Brusco S., Di Berardino C., Carpenter J., Giannelli S., Valassina N., Bido S., Ricci R., Castoldi V. (2020). dCas9-Based Scn1a Gene Activation Restores Inhibitory Interneuron Excitability and Attenuates Seizures in Dravet Syndrome Mice. Mol. Ther..

[16] Snowball A., Chabrol E., Wykes R.C., Shekh-Ahmad T., Cornford J.H., Lieb A., Hughes M.P., Massaro G., Rahim A.A., Hashemi K.S. (2019). Epilepsy Gene Therapy Using an Engineered Potassium Channel. J. Neurosci..

[17] Doudna J.A., Charpentier E. (2014). Genome editing. The new frontier of genome engineering with CRISPR-Cas9. Science.

[18] Swiech L., Heidenreich M., Banerjee A., Habib N., Li Y., Trombetta J., Sur M., Zhang F. (2015). In vivo interrogation of gene function in the mammalian brain using CRISPR-Cas9. Nat. Biotechnol..

[19] Gaudelli N.M., Komor A.C., Rees H.A., Packer M.S., Badran A.H., Bryson D.I., Liu D.R. (2017). Programmable base editing of A*T to G*C in genomic DNA without DNA cleavage. Nature.

[20] Anzalone A.V., Randolph P.B., Davis J.R., Sousa A.A., Koblan L.W., Levy J.M., Chen P.J., Wilson C., Newby G.A., Raguram A. (2019). Search-and-replace genome editing without double-strand breaks or donor DNA. Nature.

[21] Chen P.J., Liu D.R. (2023). Prime editing for precise and highly versatile genome manipulation. Nat. Rev. Genet..

[22] Nayak S., Herzog R.W. (2010). Progress and prospects: Immune responses to viral vectors. Gene Ther..

[23] Kantor B., McCown T., Leone P., Gray S.J. (2014). Clinical applications involving CNS gene transfer. Adv. Genet..

[24] Wykes R.C., Heeroma J.H., Mantoan L., Zheng K., MacDonald D.C., Deisseroth K., Hashemi K.S., Walker M.C., Schorge S., Kullmann D.M. (2012). Optogenetic and potassium channel gene therapy in a rodent model of focal neocortical epilepsy. Sci. Transl. Med..

[25] Hsu H.L., Brown A., Loveland A.B., Lotun A., Xu M., Luo L., Xu G., Li J., Ren L., Su Q. (2020). Structural characterization of a novel human adeno-associated virus capsid with neurotropic properties. Nat. Commun..

[26] Dimidschstein J., Chen Q., Tremblay R., Rogers S.L., Saldi G.A., Guo L., Xu Q., Liu R., Lu C., Chu J. (2016). A viral strategy for targeting and manipulating interneurons across vertebrate species. Nat. Neurosci..

[27] Staahl B.T., Benekareddy M., Coulon-Bainier C., Banfal A.A., Floor S.N., Sabo J.K., Urnes C., Munares G.A., Ghosh A., Doudna J.A. (2017). Efficient genome editing in the mouse brain by local delivery of engineered Cas9 ribonucleoprotein complexes. Nat. Biotechnol..

[28] Xiao X., Li J., Samulski R.J. (1996). Efficient long-term gene transfer into muscle tissue of immunocompetent mice by adeno-associated virus vector. J. Virol..

[29] Greenberg B., Yaroshinsky A., Zsebo K.M., Butler J., Felker G.M., Voors A.A., Rudy J.J., Wagner K., Hajjar R.J. (2014). Design of a phase 2b trial of intracoronary administration of AAV1/SERCA2a in patients with advanced heart failure: The CUPID 2 trial (calcium up-regulation by percutaneous administration of gene therapy in cardiac disease phase 2b). JACC Heart Fail..

[30] Maguire A.M., Simonelli F., Pierce E.A., Pugh E.N., Mingozzi F., Bennicelli J., Banfi S., Marshall K.A., Testa F., Surace E.M. (2008). Safety and efficacy of gene transfer for Leber’s congenital amaurosis. N. Engl. J. Med..

[31] Gregorevic P., Blankinship M.J., Allen J.M., Crawford R.W., Meuse L., Miller D.G., Russell D.W., Chamberlain J.S. (2004). Systemic delivery of genes to striated muscles using adeno-associated viral vectors. Nat. Med..

[32] Towne C., Schneider B.L., Kieran D., Redmond D.E., Aebischer P. (2010). Efficient transduction of non-human primate motor neurons after intramuscular delivery of recombinant AAV serotype 6. Gene Ther..

[33] Gao G., Vandenberghe L.H., Alvira M.R., Lu Y., Calcedo R., Zhou X., Wilson J.M. (2004). Clades of Adeno-associated viruses are widely disseminated in human tissues. J. Virol..

[34] Nathwani A.C., Tuddenham E.G., Rangarajan S., Rosales C., McIntosh J., Linch D.C., Chowdary P., Riddell A., Pie A.J., Harrington C. (2011). Adenovirus-associated virus vector-mediated gene transfer in hemophilia B. N. Engl. J. Med..

[35] Cunningham S.C., Spinoulas A., Carpenter K.H., Wilcken B., Kuchel P.W., Alexander I.E. (2009). AAV2/8-mediated correction of OTC deficiency is robust in adult but not neonatal Spf(ash) mice. Mol. Ther..

[36] Foust K.D., Nurre E., Montgomery C.L., Hernandez A., Chan C.M., Kaspar B.K. (2009). Intravascular AAV9 preferentially targets neonatal neurons and adult astrocytes. Nat. Biotechnol..

[37] Mendell J.R., Al-Zaidy S., Shell R., Arnold W.D., Rodino-Klapac L.R., Prior T.W., Lowes L., Alfano L., Berry K., Church K. (2017). Single-Dose Gene-Replacement Therapy for Spinal Muscular Atrophy. N. Engl. J. Med..

[38] Foust K.D., Salazar D.L., Likhite S., Ferraiuolo L., Ditsworth D., Ilieva H., Meyer K., Schmelzer L., Braun L., Cleveland D.W. (2013). Therapeutic AAV9-mediated suppression of mutant SOD1 slows disease progression and extends survival in models of inherited ALS. Mol. Ther..

[39] Yang B., Li S., Wang H., Guo Y., Gessler D.J., Cao C., Su Q., Kramer J., Zhong L., Ahmed S.S. (2014). Global CNS transduction of adult mice by intravenously delivered rAAVrh.8 and rAAVrh.10 and nonhuman primates by rAAVrh.10. Mol. Ther..

[40] Sadelain M. (2004). Insertional oncogenesis in gene therapy: How much of a risk?. Gene Ther..

[41] Li C., Samulski R.J. (2020). Engineering adeno-associated virus vectors for gene therapy. Nat. Rev. Genet..

[42] Miron-Barroso S., Domenech E.B., Trigueros S. (2021). Nanotechnology-Based Strategies to Overcome Current Barriers in Gene Delivery. Int. J. Mol. Sci..

[43] Witzigmann D., Kulkarni J.A., Leung J., Chen S., Cullis P.R., van der Meel R. (2020). Lipid nanoparticle technology for therapeutic gene regulation in the liver. Adv. Drug Deliv. Rev..

[44] Dilliard S.A., Cheng Q., Siegwart D.J. (2021). On the mechanism of tissue-specific mRNA delivery by selective organ targeting nanoparticles. Proc. Natl. Acad. Sci. USA.

[45] Francia V., Schiffelers R.M., Cullis P.R., Witzigmann D. (2020). The Biomolecular Corona of Lipid Nanoparticles for Gene Therapy. Bioconjug. Chem..

[46] Cheng M.H.Y., Brimacombe C.A., Verbeke R., Cullis P.R. (2022). Exciting Times for Lipid Nanoparticles: How Canadian Discoveries Are Enabling Gene Therapies. Mol. Pharm..

[47] Ramamoorth M., Narvekar A. (2015). Non viral vectors in gene therapy—An overview. J. Clin. Diagn. Res..

[48] Hidai C., Kitano H. (2018). Nonviral Gene Therapy for Cancer: A Review. Diseases.

[49] Jinek M., Chylinski K., Fonfara I., Hauer M., Doudna J.A., Charpentier E. (2012). A programmable dual-RNA-guided DNA endonuclease in adaptive bacterial immunity. Science.

[50] Guan Q., Wang Z., Zhang K., Liu Z., Zhou H., Cao D., Mao X. (2024). CRISPR/Cas9-mediated neuronal deletion of 5-lipoxygenase alleviates deficits in mouse models of epilepsy. J. Adv. Res..

[51] Komor A.C., Kim Y.B., Packer M.S., Zuris J.A., Liu D.R. (2016). Programmable editing of a target base in genomic DNA without double-stranded DNA cleavage. Nature.

[52] Chemello F., Chai A.C., Li H., Rodriguez-Caycedo C., Sanchez-Ortiz E., Atmanli A., Mireault A.A., Liu N., Bassel-Duby R., Olson E.N. (2021). Precise correction of Duchenne muscular dystrophy exon deletion mutations by base and prime editing. Sci. Adv..

[53] Bock D., Rothgangl T., Villiger L., Schmidheini L., Matsushita M., Mathis N., Ioannidi E., Rimann N., Grisch-Chan H.M., Kreutzer S. (2022). In vivo prime editing of a metabolic liver disease in mice. Sci. Transl. Med..

[54] Matuszek Z., Arbab M., Kesavan M., Hsu A., Roy J.C.L., Zhao J., Yu T., Weisburd B., Newby G.A., Doherty N.J. (2025). Base editing of trinucleotide repeats that cause Huntington’s disease and Friedreich’s ataxia reduces somatic repeat expansions in patient cells and in mice. Nat. Genet..

[55] Zhang H., Li Y., Li J., Li X., Li T. (2025). Base and Prime Editing for Inherited Retinal Diseases: Delivery Platforms, Safety, Efficacy, and Translational Perspectives. Pharmaceutics.

[56] Tanenhaus A., Stowe T., Young A., McLaughlin J., Aeran R., Lin I.W., Li J., Hosur R., Chen M., Leedy J. (2022). Cell-Selective Adeno-Associated Virus-Mediated SCN1A Gene Regulation Therapy Rescues Mortality and Seizure Phenotypes in a Dravet Syndrome Mouse Model and Is Well Tolerated in Nonhuman Primates. Hum. Gene Ther..

[57] Gotzsche C.R., Nikitidou L., Sorensen A.T., Olesen M.V., Sorensen G., Christiansen S.H., Angehagen M., Woldbye D.P., Kokaia M. (2012). Combined gene overexpression of neuropeptide Y and its receptor Y5 in the hippocampus suppresses seizures. Neurobiol. Dis..

[58] Ledri L.N., Melin E., Christiansen S.H., Gotzsche C.R., Cifra A., Woldbye D.P., Kokaia M. (2016). Translational approach for gene therapy in epilepsy: Model system and unilateral overexpression of neuropeptide Y and Y2 receptors. Neurobiol. Dis..

[59] Melin E., Nanobashvili A., Avdic U., Gotzsche C.R., Andersson M., Woldbye D.P.D., Kokaia M. (2019). Disease Modification by Combinatorial Single Vector Gene Therapy: A Preclinical Translational Study in Epilepsy. Mol. Ther. Methods Clin. Dev..

[60] McCown T.J. (2006). Adeno-associated virus-mediated expression and constitutive secretion of galanin suppresses limbic seizure activity in vivo. Mol. Ther..

[61] Natarajan G., Leibowitz J.A., Zhou J., Zhao Y., McElroy J.A., King M.A., Ormerod B.K., Carney P.R. (2017). Adeno-associated viral vector-mediated preprosomatostatin expression suppresses induced seizures in kindled rats. Epilepsy Res..

[62] Sorensen A.T., Nikitidou L., Ledri M., Lin E.J., During M.J., Kanter-Schlifke I., Kokaia M. (2009). Hippocampal NPY gene transfer attenuates seizures without affecting epilepsy-induced impairment of LTP. Exp. Neurol..

[63] Szczygiel J.A., Danielsen K.I., Melin E., Rosenkranz S.H., Pankratova S., Ericsson A., Agerman K., Kokaia M., Woldbye D.P.D. (2020). Gene Therapy Vector Encoding Neuropeptide Y and Its Receptor Y2 for Future Treatment of Epilepsy: Preclinical Data in Rats. Front. Mol. Neurosci..

[64] Young D., Fong D.M., Lawlor P.A., Wu A., Mouravlev A., McRae M., Glass M., Dragunow M., During M.J. (2014). Adenosine kinase, glutamine synthetase and EAAT2 as gene therapy targets for temporal lobe epilepsy. Gene Ther..

[65] Kanter-Schlifke I., Georgievska B., Kirik D., Kokaia M. (2007). Seizure suppression by GDNF gene therapy in animal models of epilepsy. Mol. Ther..

[66] Peterson A.R., Binder D.K. (2019). Post-translational Regulation of GLT-1 in Neurological Diseases and Its Potential as an Effective Therapeutic Target. Front. Mol. Neurosci..

[67] Ohno Y. (2018). Astrocytic Kir4.1 potassium channels as a novel therapeutic target for epilepsy and mood disorders. Neural Regen. Res..

[68] Lee Y., Messing A., Su M., Brenner M. (2008). GFAP promoter elements required for region-specific and astrocyte-specific expression. Glia.

[69] Noe F., Pool A.H., Nissinen J., Gobbi M., Bland R., Rizzi M., Balducci C., Ferraguti F., Sperk G., During M.J. (2008). Neuropeptide Y gene therapy decreases chronic spontaneous seizures in a rat model of temporal lobe epilepsy. Brain.

[70] Dong C., Zhao W., Li W., Lv P., Dong X. (2013). Anti-epileptic effects of neuropeptide Y gene transfection into the rat brain. Neural Regen. Res..

[71] Shimazaki K., Kobari T., Oguro K., Yokota H., Kasahara Y., Murashima Y., Watanabe E., Kawai K., Okada T. (2019). Hippocampal GAD67 Transduction Using rAAV8 Regulates Epileptogenesis in EL Mice. Mol. Ther. Methods Clin. Dev..

[72] Yan M., Zhang S., Li C., Liu Y., Zhao J., Wang Y., Yang Y., Zhang L. (2021). 5-Lipoxygenase as an emerging target against age-related brain disorders. Ageing Res. Rev..

[73] Ginn S.L., Amaya A.K., Alexander I.E., Edelstein M., Abedi M.R. (2018). Gene therapy clinical trials worldwide to 2017: An update. J. Gene Med..

[74] Hocquemiller M., Giersch L., Audrain M., Parker S., Cartier N. (2016). Adeno-Associated Virus-Based Gene Therapy for CNS Diseases. Hum. Gene Ther..

[75] Chen B., Hu J., Almeida R., Liu H., Balakrishnan S., Covill-Cooke C., Lim W.A., Huang B. (2016). Expanding the CRISPR imaging toolset with Staphylococcus aureus Cas9 for simultaneous imaging of multiple genomic loci. Nucleic Acids Res..

[76] Kim E., Koo T., Park S.W., Kim D., Kim K., Cho H.Y., Song D.W., Lee K.J., Jung M.H., Kim S. (2017). In vivo genome editing with a small Cas9 orthologue derived from Campylobacter jejuni. Nat. Commun..

